# Epigenetic alterations in gastric cancer (Review)

**DOI:** 10.3892/mmr.2015.3816

**Published:** 2015-05-22

**Authors:** DU-GUAN FU

**Affiliations:** Department of Cardiology, Xiangyang Hospital Affiliated to Hubei University of Medicine, Xiangyang, Hubei 441000, P.R. China

**Keywords:** epigenetics, gastric carcinoma

## Abstract

Gastric cancer is one of the most common types of cancer and the second most common cause of cancer-related mortality worldwide. An increasing number of recent studies have confirmed that gastric cancer is a multistage pathological state that arises from environmental factors; dietary factors in particulary are considered to play an important role in the etiology of gastric cancer. Improper dietary habits are one of the primary concerns as they influence key molecular events associated with the onset of gastric carcinogenesis. In the field of genetics, anticancer research has mainly focused on the various genetic markers and genetic molecular mechanisms responsible for the development of this of this disease. Some of this research has proven to be very fruitful, providing insight into the possible mechamisms repsonsible for this disease and into possible treatment modalities. However, the mortality rate associated with gastric cancer remains relatively high. Thus, epigenetics has become a hot topic for research, whereby genetic markers are bypassed and this research is directed towards reversible epigenetic events, such as methylation and histone modifications that play a crucial role in carcinogenesis. The present review focuses on the epigenetic events which play an important role in the development and progression of this deadly disease, gastric cancer.

## 1. Introduction

Gastric cancer is the second most common type of cancer and a leading cause of cancer-related mortality worldwide (1). An increasing number of recent studies have confirmed that gastric cancer is a multistage pathological state that arises from environmental factors; a poor diet is considered to play an imporant role in the development of this disease. Gastric cancer is classified into the intestinal and diffuse type. Differences exist in the pathways and mechanisms leading to the development of these two types of gastric cancer. Intestinal tumors develop and progress through a number of sequential steps. This begins with atrophic gastritis followed by intestinal metaplasia, dysplasia and then cancer. Diffuse gastric cancer is not characterized by any preceding steps other than chronic gastritis associated with *Helicobacter pylori* (*H. pylori*) infection (2,3).

The pathogenesis of gastric cancer represents a classic example of gene-environment interactions. Among the environmental factors, a poor (unhealthy, high-fat) diet and infection with *H. pylori* are the most common causes of gastric carcinogenesis. Genetic factors play an important role in gastric carcinogenesis due to aberrant gene expression, leading to a malignant phenotype (2). The oncogenic activation of β-catenin (17–27% in the intestinal type) and K-ras (0–18% in both histological types) has been found in gastric cancer (4,5). In addition, amplifications of the c-erbB2 and c-met genes have each been found in approximately 10% of both histological types. Among the tumor suppressor genes, p53 mutations have been reported in both the diffuse (0–21%) and intestinal type (36–43%) (6). Mutations in APC are frequently observed in gastric adenomas, but only rarely in gastric cancers (1). Somatic mutations of E-cadherin are observed specifically in sporadic diffuse type gastric cancer (33–50%) (7). Runt-related transcription factor 3 (RUNX3) has been implicated in gastric cancer, although mutations in this gene are rare (8). Microsatellite instability (MSI) is observed in 5–10% of diffuse type gastric cancer and in 15–40% of intestinal type of gastric cancer (1).

In addition to these well characterized genetic changes, epigenetic alterations, including promoter CpG island hypermethylation are the most common molecular alterations in human neoplasia (9). Promoter hypermethylation of mismatch repair gene hMLH1 is the main mechanism responsible for MSI in gastric cancer. Similarly, while the hypermethylation of p16 is common in gastric cancer with a higher incidence in the intestinal type, mutation of the p16 gene is infrequent (10). Thus, the present review focuses on the epigenetic alterations observed in gastric cancer.

## 2. Epigenetics

The term 'epigenetics' was first used by Conrad Waddington in 1939 (11) to describe 'the casual interaction between genes and their products'. Subsequently, Riggs *et al* (12) defined epigenetics as 'the study of mitotically and/or meiotically heritable changes in gene function that cannot be explained by changes in DNA sequence' (9). In the present era, the meaning of the term 'epigenetics' has broadened to include heritable and transient*/*reversible changes in gene expression that are not accompanied by a change in the DNA sequence.

The critical role of epigenetic modifications in human diseases is coming to the fore. The study of epigenetics has contribued to the comprehensive understanding of different biological activities, such as DNA methylation, chromatin structure, transcriptional activities and histone modification. Two major epigenetic modifications include DNA methylation and chromatin remodeling (9,10,13). While DNA methylation involves a chemical change in the DNA sequence that most commonly occurs at the cytosine moiety of CpG dinucleotides, chromatin remodeling occurs through histone modifications (primarily on the N-terminal tails) that ultimately affect the interaction of DNA with chromatin-modifying proteins. Both DNA methylation and histone modifications are associated with the silencing of critical tumor suppressor genes and the activation of oncogenes involved in cancer (9,10,13).

The overall 5 year survival rate for patients with gastric cancer is low (10–20%) (14). The poor prognosis associated with gastric cancer is due to the detection of the tumor at a late stage. Thus, the disovery of novel molecular markers is imperative for the early detection and prognostic prediction of this disease. The use of novel chemotherapeutic agents targeting these newly identified molecular markers may further improve the prognosis of patients with gastric cancer. Various genetic markers have been used for the early detection of tumors and prognostic prediction, as well as in an aim to elucidate the genetic pathway of gastric carcinogenesis (1,15). Epigenetic markers have gained popularity in recent years, particularly promoter hypermethylation and has various advantages over genetic markers. Firstly, promoter hypermethylation is much more common than genetic alterations in cancer. Secondly, promoter hypermethylation occurs in the same defined region of a gene in all forms of cancer in comparison to a wide range of mutational variations in a specific gene. Thus, the epigenetic detection of promoter hypermethylation may be an efficient and cost-effective method for tumor detection. Thirdly, promoter hypermethylation constitutes a 'positive signal' that can be easily detected against a background of normal cells, whereas certain genetic markers, such as the loss of heterozygosity (LOH), homozygous deletion and MSI represent 'negative signals' and are difficult to detect against a background of normal cells (16).

## 3. DNA methylation and epigenetic gene silencing

DNA methylation is a reversible chemical modification of cytosine in the CpG islands of promoter sequences, catalyzed by a family of DNA methyltransferases. DNA methylation does not alter the genetic information, but only alters the readability of the DNA and results in the inactivation of a gene by subsequent transcript repression (17). DNA methylation plays a critical role in the control of cellular process, including embryonic development, transcription, X-chromosome inactivation and genomic imprinting (18). CpG dinucleotides are not frequently observed throughout the human genome and are present at 20% of their expected frequency. Approximately half of the human gene promoter regions have CpG-rich regions of 0.5–2 kb in length where the CpG dinucleotide frequency is higher than expected. These CpG-rich sequences are often known as CpG islands (19). The majority (94%) of CpG islands remain unmethylated in a normal cell. However, particular subgroups of CpG island promoters are methylated, such as in tissue and germ line-specific genes. In general, CpG island methylation is associated with gene silencing. The methylated CpG island also recruits histone deacetylases and other factors involved in transcriptional silencing (9).

The changes in the DNA methylation status in cancer cells are complex, involving global hypomethylation and localized hypermethylation. Global hypomethylation of the genome was initially considered to be an exclusive event in cancer development (16). The loss of methylation in cancer is mainly due to the hypomethylation of repetitive DNA sequences (LINE and SINE) and the demethylation of inotropic sequences. During the development of a neoplasm, the degree of hypomethylation of genomic DNA increases as the lesion progresses from a benign one to a metastatic one (9,20). Three mechanisms of DNA hypomethylation have been suggested to be involved in the development of cancers: first, the increase in genomic instability, second by the reactivation of transposable elements, and third by the loss of imprinting. The demethylation of DNA can favour mitotic recombination, leading to deletions, translocations and chromosomal instability (9). The aberrant activation of oncogenes due to promoter demethylation (hypomethylation) has yet not been established (20). The exact association between global hypomethylation and the development of cancer remains to be consolidated (9).

Paradoxically, in parallel to global hypomethylation, the genomes of cancer cells are also characterized by localized regions of *de novo* hypermethylation, typically in the CpG island of tumor suppressor genes and microRNA (miRNA) genes. The inactivation of tumor suppressor genes through the hypermethylation of CpG islands within promoter regions is a major event in carcinogenesis (9). The hypermethylation of CpG islands also has a silencing effect on miRNAs in cancer. miRNAs are short, non-coding RNAs (18–22 nucleotides in length), that regulate a number of cellular functions, including cell proliferation, apoptosis and differentiation by silencing specific target genes through translational repression or mRNA degradation (21,22).

In the recent past, a number of genes that are critical in tumorigenesis that undergo epigenetic silencing have been identified. These include various genes involved in different cellular process, such as cell cycle regulation (p16^NK4a^, p15^INK4b^ and p14^ARF^), DNA repair [human mutL homolog 1 (hMLH1) and methylguanine DNA methyltransferase (MGMT)], cell-cell/cell matrix adhesion (E-cadherin, H-cadherin and adenomatous polyposis coli), apoptosis [death-associated protein kinase (DAPK), TMS1 and caspase-8] and angiogenesis [thrombospondin-1 (THBS-1) and p73] (16).

## 4. Histone modifications

Histones are evolutionarily highly conserved proteins characterized by an accessible aminoterminal tail and a histone fold domain that mediates interactions between histones to form the nucleosome scaffold (9). The N-terminal of histone polypeptides are extensively modified by >60 different post-translational modifications, including methylation, acetylation, phosphorylation, ribosylation, ubiquitination, sumoylation, carbonylation and glycosylation (9,13,23).

In normal cells, a precise balance maintains nucleosomal DNA in either an active/acetylated or an inactive/deacetylated form. This adequate balance is controlled by acetylating enzymes [histone acetyltransferases (HATs)] and deacetylating enzymes [histone deacetylases (HDACs)]. The other modification includes methylation of arginine and lysine residues of histones. This methylation is catalyzed by histone methyltranferase (HMT) and the process is involved in the regulation of a wide range of gene activities and chromatin structures. In general, lysine methylation at H3K9, H3K27 and H4K20 is associated with gene silencing, whereas methylation at H3K4, H3K36 and H3K79 is associated with gene activation (13). The genomic changes in the pattern of CpG methylation may in turn lead to global changes in histone modification patterns in a number of human cancers. Changes in histone modification patterns independent of CpG methylation have also been directly linked to cancer development. In addition to their role in transcriptional regulation, histone modifications have been implicated in DNA replication, repair and condensation (9).

## 5. Mechanisms of epigenetic activity

Epigenetic regulation by DNA methylation is possible through DNA methyltransferase (DNMT). In humans, various DNMTs have been identified. DNMT3A and DNMT3B have been implicated in establishing the *de novo* methylation pattern, whereas DNMT1 is considered to be responsible for maintaining the DNA methylation patterns (16,24). DNMT2 has an unknown biological function. Its strong binding to DNA suggests that it may mark a specific sequence in the genome (13).

There are three main mechanisms through which DNA methylation suppresses gene transcription (25). The first of these is methyl-CpG-binding domain (MBD)-mediated gene silencing. Various methyl-CpG-binding proteins (MBPs) have been identified, such as methyl-CpG-binding protein 2 (MeCP2) and MBD1, MBD2, MBD3 and MBD4. These proteins posses a transcriptional repressor domain, thus directly repressing transcription. In addition, MBDs can recruit transcriptional co-repressors, such as HDACs and Sin3A to methylated DNA (16,25). The deacetylation of chromatin histone results in closed or repressed chromatin configuration, which in turn leads to the exclusion of transcription factors and allele-specific gene silencing. The second mechanism of gene silencing is mediated through DNMTs. All three DNMTs (DNMT1, DNMT3A and DNMT3B) have a transcription repressor domain and can thus directly suppress transcription. In addition, these DNMTs can recruit co-transcriptional repressors, such as HDACs to methylated DNA identical to MBDs. The third mechanism is that CpG island hypermethylation can sterically interrupt the binding of activating transcription factors to gene promoters (16). The details of histone modifications are discussed above.

Polycomb group (PcG) proteins are epigenetic chromatin modifiers involved in carcinogenesis. Two distinct multiprotein PcG complexes have been identified (26,27). Polycomb repressive complex (PRC)2, which is involved in the initiation of gene repression, and PRC1 that functions as a maintenance complex. The enhancer of zeste homolog 2 (EZH2), a member of PRC2, catalyzes the addition of methyl groups to H3K27 (H3K27 trimethylation). EZH2 also interacts with DNMTs and is essential for the DNA methylation of EZH2 target promoters, suggesting that a direct link exists between PcG-mediated gene repression and DNA methylation (23).

## 6. Epigenetic alternations in gastric cancer

The majority of traditional molecular studies on gastric cancer have focused on identifying genetic mutations causing cancer or on tumor suppressor genes (28–30). However, more studies are now focusing on the discovery of novel biomarkers that are epigenetically silenced in early carcinogenesis (31–33). It has also been observed that almost half of the tumor suppressor genes that causes familial cancers through mutations are also inactivated by promoter hypermethylation in sporadic cancers (23). Increasing evidence suggests that epigenetic changes play a key role in cancer development, including gastric cancer. It has become apparent that different tumor types have a different spectrum, profile or clustering of gene hypermethylation referred to as the CpG island methylator phenotype (CIMP). The CIMP group was defined as having concordant tumor-specific DNA methylation and clearly distinguished due to exhibiting a higher methylation index in comparison to non-CIMP tumors that show only low levels of tumor-specific methylation (16).

Tumors with concurrent hypermethylation in multiple loci have been defined as CIMP-high (CIMP-H). CIMP plays an important role in the progression of gastric cancer. An *et al* (34) demonstrated that the concurrent hypermethylation of gene promoters is associated with MSI in gastric cancer with the CIMP-H phenotype. They demonstrated that the concordant methylation of multiple gene/loci found in 31% of tumors was associated with an improved survival, but was not an independent predictor of prognosis for patients with gastric cancer. Thus the prognostic role of CIMP status in gastric cancer is unclear (10).

The genes such as p16^INK4a^, CDKN2B/p15^1NK2b^ and p14^ARF^ are hypermethylated in human cell lines and primary tumors. Silencing of p16(INK4a) by promoter hypermethylation has also been reported in gastric cancer (35). The hypermethylation of p16 may predict the malignant potential of dysplasia and the early diagnosis of cancer. CDKN2A promoter methylation has been reported in 30% of gastric cancer cases (10,36). CDKN2A hypermethylation may contribute to the malignant transformation of gastric precursor lesions (37). The promoter hypermethylation of hMLH1 is a frequent event in gastric cancer and is associated with the loss of hMLH1 expression in the majority of gastric cancers exhibiting MSI (10,38–40). The methylation of hMLH1 is present in 71% of tumors with a MSI-high (MSI-H) phenotype, but only in 8% of tumors with a MSI-low (MSI-L) phenotype and in 13% of tumors with a microsatellite stable (MSS) phenotpye (37). The hypermethylation of the DNA repair protein O^6^-methylguanine DNA methyltransferase (MGMT) has been found in 31% of gastric cancer cases (41).

The capacity of cancer cells to migrate and invade other organs using vascular channels is characterized by a variety of genes, such as APC, E-Cadherin (CDH1), H-Cadherin (CDH13) and FAT tumor suppressor cadherin (13). CDH1 promoter hypermethylation has been found in 54.8% of analyzed cases of sporadic gastric cancer (42) and in 28.6 of cases, the downregulation of E-cadherin may be associated with a poor prognosis [Graziano *et al* (43)]. Of note, CDH1 promoter hypermethylation is more frequent in the diffuse histological type (18/20 cases) (43). CDH1 methylation has been shown to be significantly higher in gastric tissue with lymph node (LN) metastasis than in tissue without LN metastasis, and has been associated with serosal invasion (44). It has been reported that *H. pylori* infection is associated with E-cadherin methylation, leading to the downregulation of E-cadherin. Of note, *H. pylori* eradication therapy can reverse methylation in patients with chronic gastritis only and may halt the process of gastric carcinogenesis (10,45–47). CDH4 gene methylation has also been observed at a high frequency in gastric cancer cases and may be an early event in tumor progression (48).

The hypermethylation of DAPK has been observed in the intestinal, diffuse and mixed type of gastric cancer and correlates with the presence of LN metastasis, an advanced stage and poor survival (49). The methylation-associated inactivation of Ras association domain family 1 isoform A (RASSF1A), a tumor suppressor gene, is frequently observed in lung and breast cancer (50). The loss or downregulation of RASSF1A correlates with the stage and grade of gastric tumors. Methylation at CpG sites is observed in 95% of cases of RASSF1A non-expressing primary gastric cancer (50).

The X-linked inhibitor of apoptosis (XIAP) is the most potent member of the IAP family and exerts anti-apoptotic effects by interfering with the activities of caspases. Recently, XIAP-associated factor 1 (XAF1) has been found to negatively regulate the caspase-inhibiting activity of XIAP. The epigenetic silencing of the XAF1 gene by aberrant promoter methylation has been reported in gastric cancer (51). On the other hand, the loss of the expression of caspase-1 (interleukin-1β converting enzyme), a member of the cysteine protease family, is observed in 19.3% of cases of gastric cancer and this loss in its expression is reversed by treatment with 5-aza-2′-deoxycytidine and/or trichostatin in gastric cancer cell lines (52). The mRNA expression of TSPYL5 is frequently downregulated and inversely correlates with DNA methylation in seven out of nine gastric cancer cell lines (53). In primary gastric cancer, methylation-specific PCR of TSPYL5 has revealed hypermethylation at the CpG island in 23 out of the 36 (63.9%) cases (53). hSRBC is a putative tumor suppressor gene located at 11p15.4 and its frequent genomic loss has been observed in several malignancies. hSRBC increases the protein stability of p53 and the expression of p53 target genes, such as p21 (WAF1), PUMA and NOXA, while hSRBC-mediated cell cycle arrest and apoptosis are abolished by the blockade of p53 function. The loss or reduction of hSRBC expression has been observed in 73% of cancer cell lines and in 41% of primary gastric tumors (54). While the allelic loss or somatic mutation of this gene is infrequent, its expression is restored in tumor cells by treatment with 5-aza-2′-deoxycytidine (DNA methyltransferase inhibitor) (54). The activation of Wnt signaling has been implicated in tumorigenesis. Secreted frizzled related proteins (SFRP) are identified as possible negative modulators of the Wnt signal transduction pathway. DICKKOPF (DKK) family genes are identified as Wnt antagonists (56). The downregulated expression of SFRP2 has been shown to correlate with promoter hypermethylation in 73.3% of cases of primary gastric cancer (56). Nojima *et al* (57) found a high frequency of CpG methylation in SFRP1, SFRP2 and SFRP5 in both gastric cell lines and primary gastric cancer. The hypermethylation and loss of β-catenin (CTNNB1) expression, an integral component of the Wnt signaling pathway, has been reported in a subgroup of primary gastric cancer, cell lines and in metastases (58). DKK methylation has also reported in gastric cancer cell lines (55).

The homozygous loss of the very low density lipoprotein receptor (VLDLR) gene and epigenetic silencing by DNA methylation have been reported in gastric cancer cell lines by Takada *et al* (59). The suppressor of cytokine signaling-1 (SOCS-1) inhibits the signaling of the Janus kinase (JAK)/signal transducers and activators of transcription (STAT) pathway by several cytokines and has tumor suppressor activity. Oshimo *et al* detected the hypermethylation of the SOCS-1 gene in 44% (33/75) of cases of gastric cancer. The methylation of the SOCS-1 gene was shown to be associated with the reduced expression of the SOCS gene, LN metastases and an advanced tumor stage (60).

AKAP12/gravin is one of the A-kinase anchoring proteins (AKAPs) which functions as a kinase scaffold protein and dynamic regulator of the β2 adrenergic receptor complex. The hypermethylation of two forms of the AKAP12 gene (AKAP12A and AKAP12B) has been demonstrated in gastric cancer (61).

The promoter hypermethylation of the retinoblastoma protein-interacting zinc finger gene (RIZ1), which is involved in chromatin-mediated gene expression and is also a target for frame shift mutation in cancers with the MSI phenotype, has been shown in 69% of cases of gastric cancer and 21% of cases of non-neoplastic mucosa (62). An essential epigenetic regulator of the mammalian SWI/SNF chromatin remodeling complex contains the Brm molecule as its catalytic submit. The frequent loss of Brm expression has been observed in gastric cancer cell lines and primary gastric tumors, and this loss in its epxression is reversed by treatment with HDAC inhibitors in gastric cancer cell lines, suggesting the epigenetic regulation of this gene (63). It has been demonstrated that members of the SWI/SNF superfamily can function as tumor suppressor genes. The methylation HLTF, a homologue of SWI/SNF, has been reported in 50% of cases of gastric cancer (64).

Among the RUNX gene family, RUNX3 is often involved in gastric carcinogenesis. The hypermethylation of the CpG island of RUNX3 has been reported in 64% of cases of gastric cancer (10). Kim *et al* (65) also found RUNX3 methylation in 8% of cases of chronic gastritis, in 28% of cases of intestinal metaplasia and in 27% of cases of gastric adenoma.

The promoter hypermethylation of retinoic acid receptor β (RARβ) is observed in 64% of cases of gastric cancer (66). Thrombospondin-1 (TSP-1) is a potent peptide linked with angiogenesis in a variety of tumors. The promoter hypermethylation of the TSP1 gene is observed in 33% of cases of gastric cancer (67). The deleted in liver cancer (DLC-1) gene has been founed to be hypermethylated in 30% of cases of primary gastric cancer (68). The aberrant methylation of COX-2 has also been observed in gastric cancer (69,70).

Poplawski *et al* (71) observed aberrant methylation in the promoter regions of multiple genes (CASP8, hMLH1, CDH1 and MDR1) involved in gastric cancer. Of note, the hypermethylation of hMLH1 occurred more frequently in females than in males (71). Lee *et al* (72) reported the promoter methylation of DAPK, E-cadherin, GSTP1, p15 and p16 in 70, 76, 18, 69 and 67% of gastric cancer cases, respectively. Kang *et al* (73) investigated the methylation of multiple genes in gastric cancer tissue, gastric adenoma, intestinal metaplasia and chronic gastritis. Five different classes of methylation behaviours were observed: i) genes methylated in gastric cancer only (GSTP1 and RASSF1A), ii) genes showing a significantly higher methylation frequency in gastric cancer than in other lesions (COX-2, hMLH1 and p16), iii) genes with a high and similar methylation frequency in all four lesions (APC and E-cadherin), iv) a gene with a low and similar methylation frequency in four-step lesions (MGMT), and v) genes showing an increasing methylation frequency during the progression of the disease (DAPK, p14, THBS1 and TIMP-3). The authors concluded that tumor-related genes show a gene type-specific methylation profile along multistep carcinogenesis. Oue *et al* (74) demonstrated CpG island hypermethylation of the p16(INK4a), CDH1 and RARβ promoters in 27, 58 and 53% of gastric cancer cases, respectively. In their study, the hypermethylation of the p16 (INK4a) promoter was more common in intestinal type than in diffuse type gastric cancer. CDH1 and RARβ promoter hypermethylation was more frequently observed in the diffuse scattered type of gastric cancer (74).

Hypomethylation also contributes to gastric carcinogenesis. The demethylation of melanoma antigen (MAGE), synuclein-gamma (SNCG) and cyclin D2 has been observed in gastric cancer (10). MAGE expression is known to be activated by promethylation. The demethylation of both the MAGE-A1 and A3 promoters is more frequently observed (29 and 66%, respectively) in the advanced clinical stages of gastric cancer and is also associated with a poor prognosis (75). SNCG demethylation is common in cases with LN metastasis (76). The hypomethylation of the cyclin D2 promoter is observed in 71% of cases of gastric cancer. The hypomethylation of cyclin D2 is more common in stage III and IV tumors than in stage I and II tumors (77).

The modification of histone by methylation, which occurs at lysine or arginine residues, is generally associated with gene inactivation or silencing (78–82). Histone modifications also regulate genes that participate in the cell cycle. It has also been reported that methylation of histone H3 plays an important role in carcinogenesis by silencing tumor suppressor genes (83,84). Park *et al* (78) observed global histone modification patterns using immunohistochemistry and reported that the trimethylation of H3K9 positively correlates with tumor stage and lymphovascular invasion in gastric cancer (78). On the other hand, the acetylation of histone, which occurs mostly at lysine residues of N-terminal domains, is known to be associated with transcriptional activation. The acetylation of histone H3 at K9 has been shown to be associated with a poorly differentiated or diffuse type of histology (78). Histone H4 acetylation is reduced in gastric cancer compared to normal mucosa. The reduction of histone H4 acetylation correlates with a more advanced stage, deeper invasion and a greater extent of LN metastasis (85). In gastric cancer cells, p21^WAF1^ is associated with extensive histone acetylation. It has also been reported that reduced histone H3 acetylation is associated with reduced tumor suppressor gene p21^WAF1/CIP1^ expression in gastric cancer (86). Histone H4 acetylation at lysine 16 directs the tumor towards a better prognosis, possibly by activating tumor suppressor genes (78). Xia *et al* (87) investigated the modulation of the cell cycle control protein p21 (WAF1) by *H. pylori* in gastric cancer cells and primary gastric cells derived from healthy tissue. Their study revealed that the increased expression of p21 (WAF1) induced by *H. pylori* was associated with the release of HDAC-1 from the p21 (WAF1) promoter and the hyperacetylation of histone 4 (87).

## 7. Clinical implications of epigenetics

The knowledge of epigenetic alterations could be potentially useful for cancer diagnosis and treatment. Aberrant promoter methylation occurs very early during carcinogenesis. CpG island hypermethylation may become one of the most promising biomarkers for the early detection of tumors. It may prove to be more beneficial than genetic studies, as promoter methylation occurs more frequently in tumors than genetic alterations in general and also due to the fact that several methylated loci may be analyzed simultaneously. Since promoter methylation occurs within a well defined region of a gene, epigenetic studies are more efficient and cost-effective. The overall survival rate for patients with gastric cancer is poor (10–20%) (14). The early detection of lesions and/or reliable biomarkers for monitoring locoregional recurrence may increase the survival of patients with gastric cancer.

Epigenetic alterations in tumors may also be utilized in predicting tumor behaviour or prognosis of gastric cancer. The promoter hypermethylation of E-cadherin is associated with a poor prognosis (43). CDH1 promoter hypermethylation is more frequent in the diffuse histological type and is significantly higher with LN metastasis and serosal invasion (44,88). DAPK correlates with the presence of LN metastasis, an advanced stage and poor survival (49). The methylation of SOCS-1 leads to a with reduced expression of the SOCS gene, LN metastases and an advanced tumor stage (60). The demethylation of both the MAGE-A1 and A3 promoters is observed in advanced clinical stages and is associated with a poor prognosis (75). SNCG demethylation is common in cases with LN metastasis. The trimethylation of H3K9 in gastric cancer is associated with an advanced tumor stage and lymphovascular invasion and the acetylation of histone H3 at K9 is present in the poorly differentiated or diffuse type of histology (79). The reduction of histone H4 acetylation correlates with a more advanced stage, deeper invasion and a greater extent of LN metastasis (85).

The recent development in the understanding of relevant gene silencing by epigenetic mechanisms in cancer development is closely linked to epigenetic drug design and development. These compounds function in three processes: DNA cytosine methylation, histone modification and nucleosomal remodeling. The two main classes of drugs are DNA methylation inhibitors and HDAC inhibitors (13). The various drugs which are widely used in clinical practice for the treatment of other conditions, such as hydralazine (hypertension), procainamide (cardiac arrhythmia), valproic acid (epilepsy) may now be used for the treatment of cancer (89–91).

The use of DNA methylation inhibitors is an attractive approach for the treatment of cancer, as the toxicity of these drugs to normal cells is potentially lower than that of conventional anticancer chemotherapeutic agents (16). DNA methylation inhibitors are divided into nucleoside and non-nucleoside analogues. The former are compounds that form a covalent intermediate complex with DNMT, preventing the cell from being methylated correctly. DNMT inhibitors, such as 5-azacytidine (Vidaza), 5-aza-deoxycytidine (decitabine) are the only two cytidine analogues that have been approved by the US Food and Drug Administration (FDA) for haematological malignancies (92,93). Non-nucleoside analogues have an advantage over the analogues since they bind to the catalytic site of the enzyme DNMT and are not integrated into the DNA. Thus, this avoids the non-specific effects of nucleoside analogues (13). Hydralazine, a potent peripheral vasodilator, is currently also used as demethylating agent in cervical cancer (94).

HDAC inhibitors are divided into four groups: short chain fatty acids, hyroxamic acids, cyclic tetrapeptides and benzamides (13). The hydroxamic acid, trichostatin A (TSA), has been shown to be a chemosensitizer which increases the efficacy of chemotherapeutic drugs in gastric cancer (95). TSA is a promising chemotherapeutic agent in combination with 5-fluorouracil, paclitaxel and irinotecan in gastric cancer cells (95). Previously, (in October 2006) the FDA approved the first HDAC inhibitor, vorinostat 1 (SAHA) for the treatment of cutaneous T cell lymphoma (96). Various other HDAC inhibitors are currently undergoing clinical trials (97,98).

Another novel method for the use of epigenetics in the treatment of cancer is the reactivation of key enzymes controlling the cellular response to anticancer drugs. Satoh *et al* (99) demonstrated that microtubule inhibitors, such as docetaxel and paclitaxel induce apoptosis in gastric cells with checkpoint with forkhead-associated and ring finger (CHFR) methylation. They found that gastric cancer cells not expressing CHFR lack a mitotic checkpoint and are highly susceptible to microtubule inhibitors. Thus, CHFR methylation may be a useful molecular marker to predict the responsiveness of gastric cancer to treatment with microtubule inhibitors. Koga *et al* (100) also reported CHFR methylation in predicting the response of microtubule inhibitors in the treatment of gastric cancer.

Epigenetics may be used in the future as a tool for the discovery of potential screening markers for the early detection of gastric cancer. Epigenetics studies may also be used as a risk assessment tool for the identification of individuals at risk of developing cancer. Epigenetics may be also used in clinical practice to predict tumor behaviour and for the prognosis of patients with gastric cancer, as well as for the identification of biomarkers to monitor the response to therapeutic agents. The other application of epigenetics is in therapy. DNA methylation inhibitors and HDAC inhibitors may be used as monotherapy or in combination with other anticancer drugs for the treatment of gastric cancer.
